# Access to Fertility Preservation Counselling for Young Women with Haematological Malignancies: Incidence-Adjusted Trends from the Italian PreFerIta Network (2015–2023)

**DOI:** 10.3390/jcm15030960

**Published:** 2026-01-25

**Authors:** Renato Seracchioli, Michele Miscia, Diego Raimondo, Rossella Vicenti, Valentina Immediata, Annamaria Baggiani, Gianluca Gennarelli, Rocco Rago, Cristina Fabiani, Gemma Paciotti, Roberta Corno, Paola Anserini, Claudia Massarotti, Enrico Papaleo, Valeria Stella Vanni, Edgardo Somigliana, Francesca Filippi, Giulia Scaravelli, Lucia Speziale, Simone Bolli, Roberto De Luca

**Affiliations:** 1 Division of Gynecology and Human Reproduction Physiopathology, IRCCS Azienda Ospedaliero—Universitaria di Bologna, 40138 Bologna, Italy; renato.seracchioli@unibo.it (R.S.); rossella.vicenti@unibo.it (R.V.); 2 Department of Medical and Surgical Sciences (DIMEC), University of Bologna, 40126 Bologna, Italy; 3 Department of Gynecology, Division of Gynecology and Reproductive Medicine, Fertility Center, Humanitas Research Hospital, IRCCS, 20089 Milan, Italy; valentina.immediata@humanitas.it (V.I.); annamaria.baggiani@humanitas.it (A.B.); 4 Reproductive Medicine and IVF Unit, S. Anna Hospital University of Torino, 10126 Torino, Italy; gennarelligl@gmail.com; 5 Reproductive Pathophysiology and Andrology Unit, Sandro Pertini Hospital, 00157 Rome, Italy; rocco.rago@aslroma2.it (R.R.); cristina.fabiani@aslroma2.it (C.F.); gemma.paciotti@aslroma2.it (G.P.); roberta.corno@aslroma2.it (R.C.); 6 Physiopathology of Human Reproduction Unit, IRCCS Ospedale Policlinico San Martino, 16132 Genova, Italy; paola.anserini@hsanmartino.it (P.A.); claudia.massarotti@gmail.com (C.M.); 7 Department of Neurosciences, Rehabilitation, Ophthalmology, Genetics and Maternal and Child Health (DINOGMI), University of Genova, 16126 Genova, Italy; 8 Centro Scienze della Natalità, IRCCS Ospedale San Raffaele, 20132 Milan, Italy; papaleo.enrico@hsr.it (E.P.); vanni.valeria@hsr.it (V.S.V.); 9 Dip.to Area Materno Infantile, Fondazione IRCCS Ca’ Granda Ospedale Maggiore Policlinico, 20122 Milan, Italy; edgardo.somigliana@policlinico.mi.it (E.S.); francesca.filippi@policlinico.mi.it (F.F.); 10 ART Italian National Register, National Centre for Diseases Prevention and Health Promotion, National Health Institute, 00161 Rome, Italy; giuliascaravelli58@gmail.com (G.S.); lucia.speziale@iss.it (L.S.); simone.bolli@iss.it (S.B.); roberto.deluca@iss.it (R.D.L.)

**Keywords:** fertility preservation (FP), oncofertility, adolescents and young adults (AYA), haematological malignancies, ovarian tissue cryopreservation (OTC), oocyte cryopreservation, Italy, in vitro fertilisation (IVF), assisted reproductive technology (ART), PreFerIta

## Abstract

**Background:** Preserving fertility in young women with cancer is crucial for comprehensive care. Based on GBD 2023 estimates, approximately 1000 women aged 15–39 are diagnosed with haematological malignancies annually in Italy. Guidelines recommend timely fertility preservation (FP) counselling for all at-risk patients, yet real-world access data remain limited. **Methods:** This multicentre, retrospective observational study analysed FP counselling for women aged 15–39 with haematological malignancies from 2015 to 2023. Counselling data were extracted from the Italian Assisted Reproductive Technology Registry (IARTR). This data collection system, known as PreFerIta, was developed within a project supported by the Italian Ministry of Health to collect data on Fertility Preservation (FP) treatments in oncology patients and/or those at risk of iatrogenic infertility, provided in seven specialised ART centres across Italy. The PreFerIta database includes data on both oocyte cryopreservation and ovarian tissue cryopreservation. Annual visits were related to the estimated regional incidence of new haematological malignancies (GBD 2023). Counselling-to-incidence ratios, absolute/relative gaps, and 95% confidence intervals (CIs) were calculated. **Results:** From 2015 to 2023, an estimated 4473 new haematological malignancies occurred in the catchment regions. Concurrently, 1200 FP counselling visits were recorded. While incidence modestly declined, counselling activity remained high. The counselling-to-incidence ratio increased from 17.33% in 2015 to 31.92% in 2018, stabilising between 26% and 31% thereafter (30.98% in 2023). The relative counselling gap decreased from 82.67% to 69.02%. These ratios represent lower-bound estimates of access to specialised oncofertility consultations. **Conclusions:** In this Italian network, approximately one in four to one in three incident haematological malignancies in young women were associated with specialised FP counselling. This reflects a substantial integration of oncofertility services into haematology care, highlighting opportunities to further strengthen referral pathways and achieve full guideline concordance.

## 1. Introduction

Preserving fertility in young women diagnosed with cancer is a crucial aspect of comprehensive cancer care, with a profound impact on quality of life and future reproductive choices [1,2,3,4]. The impact of a cancer diagnosis on a young woman’s life is multifaceted. It involves not only the physical and emotional burden of the disease itself but also the distress caused by the potential loss of fertility due to the gonadotoxic effects of chemotherapy, radiotherapy, and surgery. The possibility of having children is a fundamental aspiration for many women, and the threat of infertility can lead to significant psychological distress, including anxiety, depression, and relationship difficulties [5,6]. According to recent World Health Organization projections, the global cancer burden is expected to rise substantially over the coming decades, with over 35 million new cancer cases predicted in 2050 based on IARC GLOBOCAN estimates, corresponding to an approximately 77% increase compared with 2022 levels [7,8]. Importantly, this increase is not expected to be evenly distributed across world regions and development settings, with the greatest proportional rises projected in lower- and medium-development contexts [7]. This projected growth further underscores the need to ensure timely and equitable access to fertility preservation counselling and services for young women, for whom established fertility preservation techniques offer the possibility of biological parenthood even after potentially gonadotoxic cancer treatments [1,2,3].

Among malignancies affecting women of reproductive age, haematological cancers represent a particularly high-priority setting for fertility preservation. Based on GBD 2023 estimates, approximately 1000 women aged 15–39 are diagnosed with a haematological neoplasm each year in Italy [9]. Acute leukaemias, lymphomas, and other haematological malignancies frequently occur in adolescents and young adults and are commonly treated with highly gonadotoxic regimens, including alkylating agents, high-dose chemotherapy, total body irradiation, and haematopoietic stem cell transplantation [10,11,12,13,14,15,16]. It is well established that some of these treatments are associated with a substantial risk of premature ovarian insufficiency and infertility. Furthermore, timely referral for fertility preservation is particularly challenging in this group due to the frequent need for urgent therapy initiation and specific safety concerns, particularly the risk of reintroducing malignant cells associated with ovarian tissue cryopreservation in leukaemia [10,11,12,13,14,15,16].

International guidelines, including those from the European Society of Human Reproduction and Embryology (ESHRE) and the American Society of Clinical Oncology (ASCO), emphasise the importance of informing all young cancer patients about their fertility preservation options before initiating gonadotoxic therapies [1,3,4,6]. These guidelines recommend that patients at risk of treatment-related infertility receive timely counselling and, when appropriate, be offered access to established fertility preservation procedures, such as oocyte or ovarian tissue cryopreservation [1,3,6]. Despite the availability of effective fertility preservation procedures, access to counselling and fertility preservation services has historically been variable and, in many settings, suboptimal [5,6,17,18,19,20,21]. Several factors contribute to this, including a lack of awareness and knowledge among healthcare professionals, heterogeneous availability of specialised centres, and the absence, until recently, of fully standardised national protocols for referral and treatment [5,6,17,18,19,20,21]. Moreover, much of the existing literature has focused on absolute numbers of referrals or fertility preservation procedures, without adjusting activity for underlying cancer incidence or fully tracing the entire counselling pathway [5,6,17,18,19,20,21,22,23,24]. In this context, the PreFerIta initiative was developed to systematically collect data to define trends on fertility preservation counselling and procedures provided by a network of Italian assisted reproduction centres operating within the Italian National Health Service (Servizio Sanitario Nazionale, SSN), either as public providers or as SSN-accredited institutions. The present study focuses on young women with haematological malignancies in the regions where the PreFerIta centres are located and relates the number of specialised fertility counselling visits to region-specific cancer incidence, providing incidence-adjusted indicators of access to specialised oncofertility care in this patient population using GBD 2023 estimates [9].

## 2. Materials and Methods

### 2.1. Study Design and Setting

This was a retrospective, descriptive study based on aggregated data from seven Italian public and SSN-accredited assisted reproduction centres participating in the PreFerIta network. The centres operate within public hospitals or university hospitals located in Northern and Central Italy, providing fertility preservation services to patients referred from oncological and haematological units.

### 2.2. Fertility Counselling Data

Data on the number of counselling sessions for fertility preservation were extracted from the Italian Assisted Reproductive Technology Registry (IARTR) database. This data collection system, known as PreFerIta, was developed within a project supported by the Italian Ministry of Health to systematically record fertility preservation (FP) treatments in oncology patients and those at risk of iatrogenic infertility provided in specialised ART centres across Italy. The database includes data on both oocyte and ovarian tissue cryopreservation. Embryo cryopreservation could not be included because embryo cryopreservation for fertility preservation is prohibited by law in Italy [25]. For the purposes of this analysis, we extracted annual counts (2015–2023) of consultations for women who met the following inclusion criteria: (i) age 15–39 years at the time of consultation; (ii) a diagnosis of haematological malignancy (including Hodgkin lymphoma, non-Hodgkin lymphoma, acute and chronic leukaemias, multiple myeloma, and other malignant haematopoietic neoplasms at risk of gonadotoxic therapy); and (iii) referral for potential fertility preservation in the context of anticipated or ongoing gonadotoxic treatment.

The consultations recorded in PreFerIta correspond to visits performed by reproductive medicine specialists in assisted reproduction centres, during which gonadotoxic risk is assessed, fertility preservation options are discussed, and, when appropriate, preservation procedures are planned. The collected data refer exclusively to counselling sessions and not to the number of specific FP procedures subsequently performed. While highly representative of the public-sector offer for fertility preservation in oncology within the involved regions, these data do not capture counselling activity conducted exclusively within oncology or haematology units, nor activity from private settings or public assisted reproduction centres outside the PreFerIta network. All data were anonymised and aggregated at the institutional level, with no individual patient identifiers.

### 2.3. Cancer Incidence Estimates

The Global Burden of Disease (GBD) 2023 Results Tool is a large-scale epidemiological platform developed by the Institute for Health Metrics and Evaluation (IHME) [9]. To estimate the incidence of haematological malignancies in the 15–39-year age group, GBD combines data from cancer registries, mortality statistics, ICD-coded administrative sources, longitudinal studies, and national health information systems. Within the standardised GBD estimation framework, which incorporates Bayesian modelling and ensemble approaches, cancer incidence is informed by mortality estimates and modelled mortality-to-incidence ratios (MIRs), integrating multiple sources including population-based cancer registries and vital registration systems [9].

For this study, data extraction was performed by directly querying the GBD Results Tool, version 2023 (Institute for Health Metrics and Evaluation—IHME, Seattle, WA, USA) using a standardised approach [9]. We selected the parameter “Cause of death or injury” (GBD estimate) and “Incidence” (Measure) for the metric “Number”. The query was filtered for females aged 15–39 years from 2015 to 2023 in the specific regions served by the network (Liguria, Emilia-Romagna, Piemonte, Lombardia, Lazio). The specific causes included were: Leukaemia; Hodgkin lymphoma; non-Hodgkin lymphoma; Multiple myeloma; and Myelodysplastic, myeloproliferative, and other haematopoietic neoplasms.

The choice of these categories was driven by their correspondence with the diagnoses recorded in the PreFerIta database, ensuring consistency between incidence data and the cases evaluated for fertility preservation counselling. The extraction and aggregation procedure consisted of the following steps: (1) separate extraction for each haematological category, keeping the five diagnostic groups distinct; (2) within-region aggregation of annual incidences across the selected categories to obtain total regional incidence; (3) cross-region aggregation to derive a single annual incidence estimate used as the denominator for calculating counselling-to-incidence ratios; and (4) manual recording of GBD point estimates together with their lower and upper uncertainty bounds, which were subsequently used to derive the standard errors required for constructing confidence intervals. The use of GBD 2023 offers several methodological advantages, including harmonised, comparable incidence estimates over time, reduction in distortions related to regional differences in cancer registry completeness, and the explicit incorporation of uncertainty into derived indicators [9].

### 2.4. Outcome Measures

For each year of the study period, the following metrics were calculated: (i) the total number of incident haematological malignancies in women aged 15–39 years in the catchment regions; (ii) the total number of fertility preservation counselling visits for haematological malignancies recorded in the seven PreFerIta centres; (iii) the counselling-to-incidence ratio (counselling rate), defined as the proportion of incident cases that resulted in a recorded counselling visit; (iv) the absolute counselling gap, defined as the difference between incidence and counselling visits; and (v) the relative counselling gap, expressed as a percentage of the incidence. Given that PreFerIta does not capture initial counselling performed exclusively in oncology/haematology units nor activity in centres outside the network, these counselling-to-incidence ratios represent conservative lower-bound indicators of access to specialised fertility preservation consultations.

### 2.5. Statistical Analysis

Data were presented as frequencies and percentages. To estimate 95% confidence intervals (CIs) of the counselling-to-incidence ratio, we assumed a Poisson distribution for the counselling counts and derived the standard error of cancer incidence from the GBD-provided uncertainty intervals [9]. We applied error propagation using a log-transformed scale via the delta method, which ensures approximate symmetry and constrains the interval within the positive domain. Line plots and histograms were used to graphically represent the data. Statistical analysis was performed with Stata 18 (StataCorp LLC, College Station, TX, USA) and R, version 4.3.2 (R Foundation for Statistical Computing, Vienna, Austria), using RStudio, version 2023.12.1 (Posit Software, PBC, Boston, MA, USA).

### 2.6. Ethical Considerations

The analysis used fully anonymised, aggregated data collected for institutional quality and research purposes, in line with applicable Italian regulations and local institutional policy. The study was conducted in accordance with the Declaration of Helsinki.

## 3. Results

### 3.1. Incidence and Counselling Activity

Between 2015 and 2023, an estimated 4473 incident haematological malignancies occurred among women aged 15–39 years in the five Italian regions where the PreFerIta centres are located, according to GBD 2023 estimates [9]. Over the same period, 1200 fertility preservation counselling visits for women with haematological malignancies were recorded in the seven participating assisted reproduction centres. Annual incidence of malignancies decreased from 623.08 cases in 2015 to 438.90 cases in 2021 and then stabilised at 442.67 and 445.44 cases in 2022 and 2023, respectively. In contrast, counselling activity increased from 108 visits in 2015 to 159 in 2018 and subsequently remained relatively stable, fluctuating between 119 and 150 visits per year, with 138 visits recorded in 2023.

### 3.2. Counselling-to-Incidence Ratios and Counselling Gaps

The counselling-to-incidence ratio rose from 17.33% in 2015 to 31.92% in 2018, remained between approximately 26% and 31% from 2017 onwards, and reached 30.98% in 2023. The absolute counselling gap decreased from 515.08 cases in 2015 to 307.44 cases in 2023, while the relative counselling gap declined from 82.67% to 69.02% over the same period. Overall, across the nine-year period, the counselling-to-incidence ratios indicated that approximately one in four to one in three incident haematological malignancies in young women in the regions considered were associated with a documented fertility preservation counselling visit in one of the seven PreFerIta centres.

**Figure 1:**
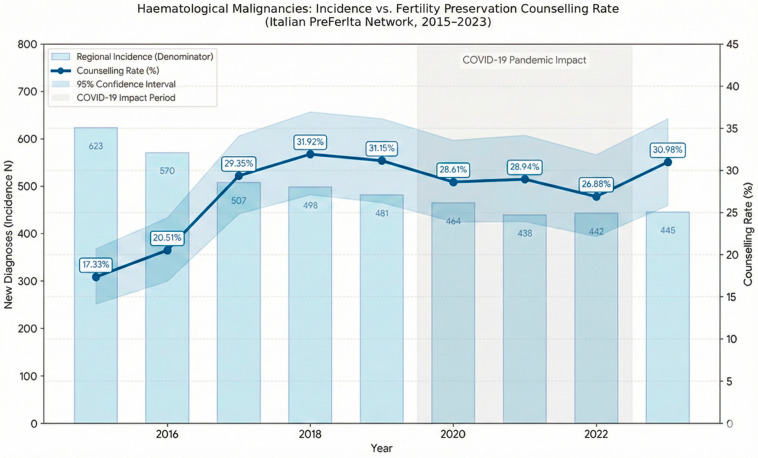
Incidence and fertility preservation counselling trends for haematological malignancies (Italian PreFerIta Network, 2015–2023). Bars (left axis): Regional incidence of new diagnoses (GBD 2023 data [9]). Line (right axis): Counselling rate (%) with 95% Confidence Intervals (shaded blue). Grey area: COVID-19 pandemic period.

Annual data are summarised in Table 1, and temporal trends for incidence, counselling visits, and counselling rate are illustrated in Figure 1.

## 4. Discussion

Haematological malignancies in young women represent a priority setting for fertility preservation, combining favourable survival prospects with a substantial risk of treatment-induced ovarian failure [10,11,12,13,14,15,16]. Given the frequent need for urgent therapy initiation and the logistical complexity of preservation strategies, rapid and structured referral pathways between haematology and reproductive medicine are essential [10,11,12,13,14,15,16].

In this study, we focused on women aged 15–39 with haematological malignancies across the PreFerIta network. By linking registry-based counselling visits to GBD 2023 regional incidence estimates, we derived incidence-adjusted trends and conservative referral proportions to benchmark our findings against international real-world data [9,10,11,12,13,14,15,16,26].

### 4.1. Distinguishing Between Levels of Counselling

Interpretation of these results requires a distinction between levels of fertility-related counselling along the care pathway [1,3,4,6]. In routine clinical practice, many patients initially receive fertility-risk information and an overview of preservation options directly from their oncologist or haematologist at diagnosis or during treatment planning [1,3,4,6]. This first-level counselling typically addresses gonadotoxicity, prognosis, timing constraints, and, in selected cases, includes preliminary discussion of fertility preservation strategies, such as GnRH analogue gonadoprotection [1,3,6]. Only a subset of patients—those desiring future pregnancy, with a prognosis compatible with long-term survival and sufficient time for intervention—are referred to a reproductive medicine unit for a specialised second-level consultation [1,3,4,6]. This specialised counselling, delivered in assisted reproduction centres, is what the PreFerIta registry captures.

Accordingly, incidence-adjusted counselling-to-incidence ratios estimate the proportion of young women with haematological malignancies who accessed specialised reproductive medicine care, not the proportion who received any fertility-related information. The observed gap between incidence and recorded consultations should therefore not be interpreted as the absence of counselling: many women may have received first-level discussions or appropriately declined referral based on age, parity, completed family size, prognosis, or personal preferences [1,3,4,6,8,9,10,11,12,13,14].

### 4.2. Interpretation of Counselling Rates and Gaps

Our analysis indicates that approximately one in four to one in three adolescents and young adults accessed a reproductive medicine specialist before initiating potentially gonadotoxic therapy [9,10,11,12,13,14,15,16]. The increase in counselling-to-incidence ratios between 2015 and 2018, followed by a plateau around 26–31%, suggests the progressive strengthening of oncofertility integration within haematology pathways across the network. Counselling volumes remained high despite declining incidence, resulting in a higher proportion of patients evaluated by reproductive specialists [9].

These estimates are consistent with haematology-focused series and reviews, in which pre-chemotherapy referral or specialist counselling rates in young women typically range from approximately 20% to just over 30%, depending on disease type, organisational setting, and time period [10,11,12,13,14,15,16]. In referred cohorts, international data indicate that oocyte and embryo cryopreservation remain the predominant strategies, with increasing use of ovarian tissue cryopreservation in selected settings and encouraging medium-term reproductive outcomes [10,11,12,13,14,15,16].

Nevertheless, in the context of international real-world data, PreFerIta counselling-to-incidence ratios, stabilising around 26–31% in recent years, appear to fall within the lower–middle range of reported performance [4,5,6,10,11,12,13,14,15,16,17,18,19,20,21,22,23,24,26]. They exceed some population-based estimates of completed oncofertility consultations but remain below the rates achieved by the best-performing structured programmes, where organisational interventions have increased documentation of fertility discussions and specialist-access indicators, in some settings exceeding 60% depending on the endpoint assessed [6,22,23,26].

The counselling gap likely reflects multiple factors, including appropriate non-referral or patient choice following first-level counselling, urgent treatment needs—particularly in acute leukaemias—and strict timing constraints [1,3,4,6,10,11,12,13,14,15,16]. In addition, it is important to underline that PreFerIta includes only seven participating assisted reproduction centres and does not capture counselling delivered in other public or private settings, leading to an underestimation of specialised counselling activity.

### 4.3. Comparisons with Other Studies and Malignancies

Real-world studies from different countries often report markedly different proportions of patients accessing oncofertility care, largely because they quantify different steps of the pathway—documentation of discussions, placement of referrals, attendance at specialist consultations, or uptake of fertility preservation procedures [4,5,6,17,19,20,21,22,23,24,26,27]. In PreFerIta, the numerator reflects attendance at a second-level specialist consultation within ART centres, while the denominator reflects population incidence of haematological malignancies [9]. Comparisons are therefore most informative when aligned to specialist-access endpoints.

This variability across studies is consistent with the broader framework outlined by major guideline harmonisation efforts. The PanCareLIFE Consortium and the International Late Effects of Childhood Cancer Guideline Harmonization Group developed evidence-based recommendations for fertility preservation in childhood, adolescent, and young adult cancer, explicitly highlighting a persistent implementation gap between evidence-based guidance and routine clinical practice [4]. In particular, they noted that patients are not always adequately counselled about fertility risks and options, nor consistently referred to fertility preservation specialists [1,3,4,6]. In one cohort included in the supplementary evidence base, reproductive specialist input on fertility preservation was documented in only 27% of patients [4], a proportion closely aligned with specialist-access estimates observed in several real-world settings.

Evidence from haematology-focused or mixed AYA cohorts further indicates that limited specialist access is not unique to the Italian setting. In a worldwide survey of haematopoietic cell transplant specialists, awareness and communication regarding fertility risks were high, with 87% reporting that they routinely informed patients; however, referral rates remained substantially lower, particularly for women (36%), indicating persistent attrition between discussion and specialist involvement even in highly specialised haematological environments [27]. This pattern mirrors the gap observed in PreFerIta between estimated incidence and recorded specialist consultations, supporting the interpretation that organisational and pathway-related factors, rather than lack of awareness alone, play a key role.

Similarly, mixed-malignancy cohorts that include a substantial proportion of haematological patients report comparable specialist-access figures when downstream steps are measured. In an Australian retrospective cohort, Chadwick et al. documented fertility preservation discussions in 58% of women but observed that only 25.9% attended a specialist gynaecology consultation, with a further 11.2% declining referral when offered [26]. The specialist-attendance proportion closely aligns with the plateau observed in PreFerIta (~27–31%). While their study includes multiple tumour types and captures upstream documentation, whereas PreFerIta begins at the specialist step and links activity to population incidence across regions, both datasets point to a similar ceiling for specialist access in the absence of fully standardised referral pathways [9,26].

Beyond patient- and disease-related factors, organisational determinants strongly influence access to fertility preservation counselling across healthcare systems [5,6,17,18,19,20,21,22,23,24]. International data consistently show higher documentation and referral rates in academic centres and multidisciplinary programmes, alongside persistent disparities related to socioeconomic status and geography [5,6,17,18,19,20,21,22,23,24]. The communication–referral gap observed among transplant specialists—where referral occurred in only 56% of male and 36% of female patients despite high rates of fertility-risk discussion—illustrates how organisational barriers can persist even in highly specialised contexts [27]. Comparable heterogeneity has been described in breast cancer cohorts in the absence of structured referral pathways, reinforcing the relevance of system-level determinants beyond tumour biology alone [19,20,21,23].

Comparisons with solid tumours provide further context. Breast cancer cohorts, which dominate the oncofertility literature, often benefit from more predictable treatment planning windows, yet they show persistent attrition along the care pathway. In a French regional population-based cohort of women aged 18–40 treated with chemotherapy for breast cancer, Martinet-Kosinski et al. reported that 41% received fertility information and 28% attended an oncofertility consultation; among those informed, uptake of consultation was high [19]. The consultation proportion is strikingly similar to that observed in PreFerIta, despite differences in tumour biology and feasibility, suggesting that specialist access rates of this magnitude recur across settings when referral is not universal.

Other breast cancer studies illustrate how endpoint definition shapes observed access. Using an administrative proxy for infertility-related consultations in Ontario, Korkidakis et al. reported lower overall proportions, increasing over time but remaining below figures derived from chart-based or registry-based specialist attendance [20]. Downstream procedure-based endpoints further emphasise attrition: in a nationwide French cohort, Duraes et al. found that fertility preservation via oocyte retrieval after ovarian stimulation reached 17% in 2019, highlighting that even when consultation occurs, eligibility, timing, and patient preferences continue to restrict uptake [21]. At the institutional level, Yuen et al. reported that although over half of reproductive-age women with breast cancer received oncofertility counselling, only about one third of those counselled were referred to reproductive endocrinology services [24].

Comparable gaps are reported in other solid tumours. In gynaecologic malignancies, fertility-sparing decisions may be partly embedded within the gynaecologic oncology pathway, yet referral to reproductive medicine remains limited. In a cohort of reproductive-age women with newly diagnosed gynaecologic cancers, Frisch et al. reported referral to REI in 14.6% of cases, with fertility desire documented in fewer than half; notably, referral was not associated with longer time to treatment [28]. In gastrointestinal cancers, Gudmundsdottir et al. identified substantial underutilisation of pretreatment fertility counselling using natural language processing of clinical records, suggesting that fertility considerations may be overlooked when they are not embedded as a default component of care [29].

International experience also highlights the value of dedicated registries in interpreting and benchmarking access to oncofertility services. PreFerIta shares conceptual similarities with initiatives such as the Japan Oncofertility Registry, which has supported service monitoring and national policy development [30,31]. Across different healthcare systems, registry data consistently show expansion of oncofertility services over the past decade, with persistent heterogeneity and incomplete access outside high-volume academic centres [9,30,31]. Linking registry activity to population-based incidence, as performed in the present study, provides a reproducible approach to benchmarking specialist access and identifying targets for intervention [9,30,31].

Across countries and malignancies, these studies converge on a consistent pattern. Specialist-level access to oncofertility care commonly involves only a minority of eligible patients, often clustering around one quarter to one third when measured as consultation attendance [19,26]. Differences between settings are shaped not only by tumour biology and urgency, but also by organisational determinants such as referral triggers, documentation practices, the availability of rapid-access services, and reimbursement policies [7,17,19,20,21,24,26,27,28]. Within this context, PreFerIta’s incidence-adjusted counselling-to-incidence ratios appear consistent with real-world specialist-access benchmarks, while leaving substantial room for improvement through system-level interventions.

### 4.4. Strengths and Limitations

Strengths of this study include the multicentre design, use of a standardised national registry for counselling activity, and integration with region-specific incidence estimates derived from external sources [9]. This approach enables calculation of incidence-adjusted counselling rates, which are more informative than absolute counts and allow longitudinal and inter-regional comparisons. Furthermore, while most published literature on real-world referral patterns has focused on breast cancer patients [17,19,20,21,22,23,24], this study provides specific data on haematological malignancies [10,11,12,13,14,15,16]. Focusing on counselling delivered within public and SSN-accredited assisted reproduction centres provides a conservative but robust estimate of access to specialised oncofertility care within a regional network [4,5,6,26].

Several limitations should be acknowledged. PreFerIta currently includes only seven public and SSN-accredited assisted reproduction centres and does not capture counselling or procedures delivered elsewhere, resulting in likely underestimation of national access. Registry data may be affected by under-reporting or miscoding, although participating centres are experienced tertiary units. Aggregated data precluded stratification by haematological subtype, age subgroups, parity, socioeconomic status, or other determinants known to influence counselling and referral [4,5,6,8,9,10,11,12,13,14,15,16,17,18,19,20,21,22,23]. Linkage to downstream reproductive outcomes was not possible and will require future integration of PreFerIta procedural and follow-up data with cancer registries. Finally, COVID-19-related disruptions may have influenced incidence estimates and referral patterns during part of the study period, although this could not be formally quantified in the present analysis [32].

## 5. Conclusions

In this Italian network, approximately one in four to one in three incident haematological malignancy cases in women aged 15–39 years were associated with a specialised fertility preservation counselling visit recorded in the participating centres. By linking registry-based counselling activity to regional incidence estimates, these counselling-to-incidence ratios provide conservative, lower-bound indicators of access to second-level oncofertility consultation across the catchment areas.

While these findings suggest a substantial integration of oncofertility services into haematology care, a persistent counselling gap remains. Further strengthening structured referral pathways, embedding fertility-related prompts and standardised procedures into haematology workflows, and ensuring timely access to specialised counselling may help increase coverage and achieve full concordance with recommendations that eligible patients receive comprehensive fertility preservation counselling.

## Figures and Tables

**Table 1:** Annual trends in fertility preservation counselling, cancer incidence, and referral gaps for women with haematological malignancies (Italian PreFerIta Network, 2015–2023).

Year	Counselling (N)	Regional Incidence (N) *	Absolute Gap (N) **	Relative Gap (%) ***	Counselling Rate (%)	95% CI ****
2015	108	623.08	515.08	82.67%	17.33%	14.12–20.70
2016	117	570.48	453.48	79.49%	20.51%	16.83–24.37
2017	149	507.59	358.59	70.65%	29.35%	24.82–34.08
2018	159	498.08	339.08	68.08%	31.92%	27.10–36.94
2019	150	481.49	331.49	68.85%	31.15%	26.17–36.14
2020	133	464.84	331.84	71.39%	28.61%	23.88–33.56
2021	127	438.90	311.90	71.06%	28.94%	23.92–34.18
2022	119	442.67	323.67	73.12%	26.88%	22.14–31.85
2023	138	445.44	307.44	69.02%	30.98%	25.82–36.14

Legend: * Regional Incidence: New diagnoses in Lombardia, Piemonte, Liguria, Emilia-Romagna, and Lazio (Source: GBD 2023 [9]). ** Absolute Gap (N): Calculated as Regional Incidence—Counselling. Represents the estimated number of women who did not receive specialised counselling. *** Relative Gap (%): Calculated as (Absolute Gap/Regional Incidence) × 100. Represents the proportion of unmet need. **** 95% CI: Confidence Intervals calculated using the delta method to account for uncertainty in cancer incidence estimates.

## Data Availability

The data presented in this study are available within the article.

## Abbreviations

The following abbreviations are used in this manuscript:

ART	Assisted Reproductive Technology
ASCO	American Society of Clinical Oncology
AYA	Adolescents and Young Adults
CI	Confidence Interval
COVID-19	Coronavirus Disease 2019
ESHRE	European Society of Human Reproduction and Embryology
FP	Fertility Preservation
GBD	Global Burden of Disease
ICD	International Classification of Diseases
IARTR	Italian Assisted Reproductive Technology Registry
IHME	Institute for Health Metrics and Evaluation
IVF	In Vitro Fertilisation
MIR	Mortality-To-Incidence Ratio
OTC	Ovarian Tissue Cryopreservation
POI	Premature Ovarian Insufficiency
SSN	Servizio Sanitario Nazionale
